# Novel renal markers for the assessment of renal integrity in patients undergoing knee arthroplasty – a pilot study

**DOI:** 10.1186/s40634-018-0159-z

**Published:** 2018-09-25

**Authors:** Annika Piirainen, Jukka Huopio, Hannu Kokki, Anu Holopainen, Teemu Pajunen, Kari Pulkki, Merja Kokki

**Affiliations:** 1 Anaesthesia and Operative Services, Kuopio University Hospital, PO Box 100, FI-70029 KYS Kuopio, Finland; 2 Department of Anaesthesiology and Intensive Care, School of Medicine, University of Eastern Finland, Kuopio, Finland; 3 Department of Orthopaedics, Traumatology and Hand Surgery, Kuopio University Hospital, Kuopio, Finland; 4 Eastern Finland Laboratory Centre Joint Authority Enterprise (ISLAB), Kuopio, Finland; 5 Laboratory Division, Turku University Hospital, Turku, Finland

**Keywords:** Arthroplasty, replacement, knee, Acute kidney injury, Neutrophil gelatinase associated lipocalin, Kidney injury molecule-1 protein, Creatinine, Glomerular filtration rate, Cystatin C, Anti-inflammatory agents, non-steroidal

## Abstract

**Background:**

The feasibility of novel kidney injury biomarkers in consecutive patients having total knee arthroplasty with local infiltration analgesia was evaluated.

**Methods:**

We enrolled 30 patients scheduled for elective unilateral total knee arthroplasty. Paired plasma and urine samples were taken before surgery and at 4 h, 24 h and 48 h after surgery to measure creatinine, cystatin C, neutrophil gelatinase associated lipocalin, kidney injury molecule-1, interleukin-18 and liver-type fatty acid-binding protein.

**Results:**

At baseline, 13 subjects had normal kidney function, 15 had mild and two had moderate kidney failure evaluated by calculated glomerular filtration rate. None of the subjects had all measured novel renal markers below proposed cut-off concentrations. Altogether 28/30 subjects had one (*n* = 3), two (*n* = 7) or three (*n* = 18) plasma neutrophil gelatinase associated lipocalin values above normal. In seven of these 28 subjects plasma creatinine, calculated glomerular filtration rate and plasma cystatin C were within the reference values. Five subjects had a low urine output, < 0.5 mL/h, indicating transient acute kidney injury, four of these had high plasma neutrophil gelatinase associated lipocalin and one high plasma cystatin C.

**Conclusions:**

In the present study plasma neutrophil gelatinase associated lipocalin was elevated in most subjects with total knee arthroplasty and local infiltration analgesia as a marker of possible renal proximal tubular injury. Five subjects had transient low urine output, but none developed renal deterioration requiring treatment.

## Background

Depending on the definition, the incidence of acute kidney injury (AKI) in patients with total knee arthroplasty (TKA) is up to 6.2% (Nowicka and Selvaraj 2016; Gharaibeh et al. 2017). This is a concern because post-operative AKI is an independent predictor of mortality (Lafrance and Miller 2010; Schlondorff 1993). The increased mortality risk persists in patients whose renal function recovers by the time of discharge from hospital (Lafrance and Miller 2010).

In compromised situation, for example hypovolemia or hypoperfusion renal homeostasis is dependent on vasodilating prostaglandins. In these conditions, blocking prostaglandin synthesis by nonsteroidal anti-inflammatory analgesic drugs (NSAID) may cause renal ischemia and predispose to acute tubular necrosis (Schlondorff 1993). Patients’ concomitant diseases, diabetes mellitus, hypertension, arteriosclerosis and cardiac insufficiency are also risk factors for AKI (Gharaibeh et al. 2017; Weingarten et al. 2012).

Use of NSAIDs is common in orthopaedic surgery because surgery causes pain and inflammation (Koppensteiner et al. 2011). Local infiltration analgesia (LIA) has gained popularity in postoperative pain management (Hu et al. 2016). The LIA solution consists of local anaesthetic, epinephrine and ketorolac. Ketorolac is one of the most potent NSAIDs. It is related to NSAID induced AKI and thus, its use is a concern in this patient population (Affas et al. 2014).

Early diagnostics of AKI is challenging. Plasma creatinine and urine output are considered the golden standards of detecting AKI (Kellum et al. 2012). However, plasma creatinine (P-crea) based approach may delay AKI diagnosis and produce false negative results (Swedko et al. 2003; Ostermann and Joannidis 2016). New tests have been developed in order to find sensitive, fast and reliable markers of renal deterioration.

Neutrophil gelatinase associated lipocalin (NGAL) is a glycoprotein that is expressed in kidney and bone marrow. In kidneys NGAL is filtrated into urine and reabsorbed in proximal tubules. In plasma NGAL increases for example in septic infections (Schrezenmeier et al. 2017). In AKI both P- and urine- (U) NGAL increase proportionally to severity of renal deterioration at early phase (2–6 h) (Haase et al. 2009). Increased P-NGAL predicts impaired outcome and increased mortality in cardiac surgery and septic infections (Haase et al. 2009, 2011). However, data about P-NGAL use in different types of surgery is sparse (Shavit et al. 2011).

Kidney injury molecule-1 (KIM-1) is highly expressed in renal proximal tubular cells in post ischaemic situations while the concentration in urine of healthy humans is low (Ichimura et al. 2004; Schrezenmeier et al. 2017). Urine-KIM-1 and U-NGAL are assumed to be highly sensitive, early markers of proximal tubular injury and thus, appropriate for perioperative use (Schrezenmeier et al. 2017).

Interleukin-18 (IL-18) is synthesised as an inactive precursor that requires caspace-1 for cleavage into an active pro-inflammatory cytokine. IL-18 is produced in the collecting ducts and is induced broadly in injured tubular epithelial cells due to development and progression of AKI (Schrezenmeier et al. 2017). However, the diagnostic accuracy of U-IL-18 in predicting AKI is still uncertain (Lin et al. 2015).

One of the most recently recognized renal biomarker, urine liver-type fatty acid-binding protein (U-L-FABP) is a small, protein expressed in human proximal tubule. Due to the small size, L-FABP is easily leaked out of damaged proximal tubular cells into the urine (Schrezenmeier et al. 2017).

Novel, sensitive renal biomarkers have been evaluated in patients with cardiac surgery and liver and kidney transplantation surgery. In major orthopaedic surgery their use has not been established. The aim of the present observational pilot study was to evaluate the feasibility of the novel, sensitive renal biomarkers, NGAL, KIM-1, IL-18 and L-FABP in consecutive patients undergoing elective, unilateral TKA in patients with LIA and postoperative NSAID/acetaminophen analgesia. Our study hypothesis was that these biomarkers would be more sensitive to detect changes in renal function than P-crea, calculated glomerular filtration rate (eGFR) and P-cystatin C in orthopaedic surgical patients. The results of the present study will be used to plan a clinical study concerning renal effects of LIA use with NSAIDS in TKA patients.

## Methods

The study was approved by the local research Ethics Committee, was undertaken according to the declaration of Helsinki. Informed consent was obtained from patients.

A total of 34 consecutive patients admitted to hospital for elective unilateral TKA were asked to participate, and 30 of them agreed. Four patients did not give any specific reasons to decline. Subjects included were 54–75 years old, and had an American Society of Anesthesiologists physical status of I-III. We did not enrol patients with body mass index < 18 kg/m^2^ or > 35 kg/m^2^, those with hepatic or severe renal impairment, P-crea > 90 μmol/L in women and > 100 μmol/L in men.

Anaesthesia was standardized. Lumbar puncture was performed at L3–4- or L4–5-interspace. Spinal anaesthesia was induced with levobupivacaine 5 mg/mL with fentanyl 10–20 μg. A urinary catheter was inserted after spinal anaesthesia, and it was removed before discharge to the surgical ward. Decrease in mean arterial pressure below 65 mmHg was treated with bolus of phenylephrine and fluid therapy according to the hospital protocol using Ringer’s acetate solution.

A standardized surgical technique was used. Tourniquet was applied in all procedures before starting the operation. A straight midline skin and medial parapatellar fascial incisions were used in approach. Patella was everted and bone cuts were made by oscillating saw utilizing intramedullar instrumentation in femur and extramedullar in tibia. Bone cement was used to fix the components. Patella was not resurfaced. Intraoperative LIA was used. Fascia and subcutaneous tissues were sutured in layers and skin was closed with staples. After draping the wound, tourniquet was deflated.

Urine/plasma samples were taken at induction of anaesthesia and at 4 h (h) and at 24 h after surgery for the measurement of P- and U-NGAL, U-KIM-1, U-L-FABP, and U-IL-18. Blood samples for measurement of P-crea and P-cystatin C were collected at preoperative visit to the hospital 1–2 weeks before surgery, at induction of anaesthesia and at 24 h and 48 h after surgery.

The intensity of post-operative pain was assessed with an 11-point numeric rating scale (NRS, 0 = no pain, 10 = most pain). Pain scores at rest and with knee movement were recorded hourly during the first four postoperative hours and at 24 h and 48 h after surgery.

Postoperative pain was treated according to the hospital guideline. LIA was used at the end of surgery. Subjects received acetaminophen 1–2 g by mouth 60 min before surgery and after that 1 g three times in 24 h (*n* = 29). NSAIDs were allowed after the first four postoperative hours; 13 subjects received first a single intravenous (i.v.) injection of ketoprofen 50–100 mg and 26 subjects received meloxicam 7.5 mg × 1–2 by mouth. Oxycodone-naloxone controlled release tablets 5/2.5 or 10/5 mg × 2 was given for all subjects and oxycodone i.v., subcutaneously or by mouth was allowed for rescue analgesia. For severe pain single shot femoral block with 10 mL of levobupivacaine 2.5 mg/mL (*n* = 14) and an epidural infusion of levobupivacaine-fentanyl-epinephrine was used (*n* = 2).

Definition of acute kidney injury was based on Kidney Disease Improving Global Outcomes (KDIGO) criteria: an increase in P-crea by ≥26.5 μmol/L within 48 h; or an increase in P-crea to ≥1.5 times compared to baseline at preoperative visit; or urine output < 0.5 mL/kg/hour for 6 h (Kellum et al. 2012).

### Sample analyses

Blood samples were collected into heparinized tubes and urine samples into polypropylene tubes. Blood samples were centrifuged with 2500 G for 15 min right after sampling and stored at − 70 °C until the analysis. Urine samples were centrifuged with 500 G for 5 min, divided in two polypropylene tubes and frozen immediately at − 70 °C until the analysis.

P-crea was analysed in hospital laboratory (http://webohjekirja.mylabservices.fi/ISLAB/) using enzymatic photometric method. eGFR was calculated using Chronic Kidney Disease Epidemiology Collaboration formula (Levey et al. 2009) (Table 1). P-cystatin C was analysed using immunochemical, photometric method in the hospital laboratory. Plasma and U-NGAL, IL-18 and KIM-1 concentrations were analysed with commercial quantitative enzyme-linked-immuno-sorbent assay (ELISA) of two-step sandwich enzyme immunoassay kits (human NGAL ELISA kit, BioPorto Diagnostics (Hellerup, Denmark); human IL-18 ELISA kit, MBL International Corporation (Woburn, MA, USA); human T cell immunoglobulin and mucin domain-1 (TIM-1)/KIM-1/hepatitis A virus cellular receptor 1 (HAVCR) Quantikine® ELISA Kit, R&D Systems, Inc. (Minneapolis, MN, USA), and L-FABP with human L-FABP Assay Kit 96 test, CMIC Co., Ltd. (Tokyo, Japan)) in the hospital laboratory. Urinary biomarker concentrations, except IL-18, are reported as normalized ratio to U-crea concentration in order to control variations in urine flow rate. Reference/cut-off values are presented in Table 1 (Human NGAL, ELISA Kit 036CE, Instruction sheet n.d.; Nisula et al. 2015; Quantikine® ELISA Human TIM-1/KIM-1/HAVCR Immunoassay n.d.; Kamijo-Ikemori et al. 2011; Devarajan 2010).

**Table 1:** Reference values for kidney injury biomarkers. Data are given as mean (minimum- maximum) or cut-off values

Parameter	Reference values/cut of value
P-NGAL	62.1 (40.8–104) ng/mL
U-NGAL	25.3 (2.4–154) ng/mL
	> 104 ng/mL ^2^
U-NGAL/crea	> 300 ng/mg·crea
U- KIM1	1.11 (0.225–3.20) μg/g·crea
U- IL-18	> 65 pg/mL
U- L-FABP	1.6 (0.3–8.4) μg/g·crea
eGFR (mL/min/1.73m^2^)	
Healthy	≥90
Mild renal deterioration	60–89
Mild to moderate	45–59
Moderate to severe	30–44
Severe	15–29
End stage	< 15
P-Crea	
Female	50–90 μmol/L
Male	60–100 μmol/L
P-cystatin C	0–1.1 mg/L

*P* plasma, *U* urine, *Crea* creatinine, *eGFR* calculated creatinine clearance, *CystC* cystatin C, *U-NGAL/crea* Neutrophil gelatinase associated lipocalin/creatinine, *KIM-1/crea* Kidney injury molecule-1, *IL*-18 Interleukin-18, *U-L-FABP/crea* liver-type fatty acid-binding protein (U-L-FABP)

After a minimum of 12 month after the surgery the patient charts were examined for subjects’ long-term renal outcome.

### Statistics

For this pilot study no formal sample size calculation was performed but a sample of 30 subjects was considered to provide sufficient information about the feasibility of these novel renal biomarkers in orthopaedic patients as to our knowledge no previous data about these sensitive renal markers in patients with TKA was available. Data were entered and analysed with the Statistical Package for Social Science -software (SPSS 22.0, IBM Corporation, Armonk, NY, USA). Because there was no control group, descriptive data (number of cases or median with minimum-maximum) are presented. Continuous and nominal variables were assessed with the Mann-Whitney U-test and the Wilcoxon Signed Rank test, where appropriate. Correlations were calculated using the Pearson’s correlation coefficient. A two-sided *P*-value of 0.05 was considered as the limit of statistical significance.

## Results

The baseline characteristics of the subjects are presented in Table 2. The fluid input during surgery and 4-h recovery room stay was for the first hour 500 mL/h then a median of 280 (minimum-maximum, 120–480) mL/h. There was one protocol deviation; one woman with preoperative P-crea of 100 μmol/L was recruited and her data was included in the final analysis. There were no drop-outs.

**Table 2:** Patients’ characteristics, data are presented as median and (minimum-maximum) or number of cases

Variable	Patients (*n* = 30)
Age (years)	66 (54–75)
Gender (female/male)	24/6
BMI (kg/m^2^)	28.5 (19.0–36.2)
Height (m)	1.6 (1.5–1.8)
Weight (kg)	75 (51–111)
Chronic diseases (yes/no)	29/1
-Hypertension (yes/no)	14/16
-Diabetes (yes/no)	8/22
ASA I/II/III	5/10/15
Preoperative pain medication (yes/no)	25/5
Surgical duration (hh:min)	0:54 (0:31–1:32)
Urine output during surgery, time at the recovery room (mL/kg/h)	1.2 (0.2–4.9)

### Traditional renal markers and urine output

Preoperative median P-crea was 65 (49–102) μmol/L and eGFR 89 (46–100) mL/min/1.73m^2^. At baseline 13 subjects had normal kidney function, 15 subjects had mildly and two women had mildly-moderate decreased renal function. At 48 h eGFR had decreased in seven out of 30 subjects; median decrease 5 (1–24) mL/min/1.73m^2^ compared to baseline, indicating mild decrease in renal function. In these seven subjects P-crea at 48 h postoperatively varied between 61 and 85 μmol/L.

At baseline P-crea was increased in one woman, 100 μmol/L, and at 48 h her P-crea was 92 μmol/L. At 48 h eight subjects had P-crea higher and 19 lower than that at baseline, median change 4 (18–22) μmol/L.

Preoperatively P-cystatin C was above 1.1 mg/L in seven subjects, at 24 h in four and at 48 h in five subjects, respectively. At 48 h five subjects with P-cystatin C > 1.1 mg/L, had eGFR ≤90 mL/min/1.73m^2^.

The median of urine output during the surgery and the first four postoperative hours was 1.2 (0.2–4.9) mL/kg/h. In five subjects perioperative urine output was less than 0.5 mL/kg/h for a median of 5.6 (5.4–6.1) h, i.e. these subjects developed a transient stage 1 AKI defined by the KDIGO urine output criteria (Levey et al. 2009).

### Novel renal biomarkers

Ten subjects had P-crea, eGFR and P-cystatin C within normal values in all three time points; preoperative, 24 h and 48 h. None of these subjects had all measured new renal markers below the upper limits of normal, P-NGAL was most often elevated (*n* = 28). In all 20 subjects with increased P-crea or P-cystatin C, or decreased eGFR, P-NGAL was significantly elevated in two (*n* = 4) or all three (*n* = 16) time points.

**Fig. 1:**
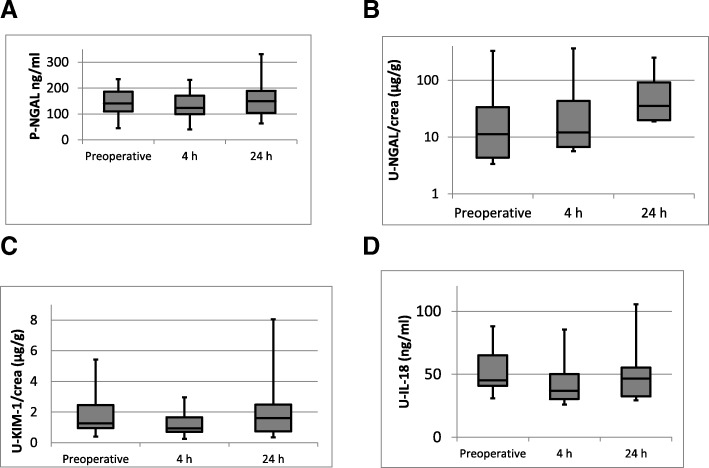
**a** Plasma neutrophil gelatinase associated lipocalin (NGAL) concentrations preoperatively, at 4 h and at 24 h after surgery presented as box and whisker plots with median, lower and upper quartiles, minimum and maximum. **b** Urine neutrophil gelatinase associated lipocalin/creatinine (U-NGAL/crea) concentration ratios preoperatively, at 4 h and at 24 h after surgery presented as box and whisker plots with median, lower and upper quartiles, minimum and maximum. **c** Urine kidney injury molecule-1/creatinine (U-KIM-1/crea) concentration ratios preoperatively, at 4 h and at 24 h after surgery presented as box and whisker plots with median, lower and upper quartiles, minimum and maximum. **d** Urine interleukin − 18 concentrations preoperatively, at 4 h and at 24 h after surgery presented as presented as box and whisker plots with median, lower and upper quartiles, minimum and maximum

P-NGAL results are presented in Fig. 1a. Moderate negative correlations were observed with preoperative and 24 h eGFR with preoperative P-NGAL (r, − 0.52 and − 0.45 (*p* < 0.05). Preoperative, 24 h and 48 h eGFR had a moderate negative correlation with P-NGAL at 4 h (− 0.46, − 0.54, − 0.42, p < 0.05) and at 24 h (− 0.56, − 0.66, − 0.54, *p* < 0.003) also. In those five subjects with decreased urine output P-NGAL was elevated in four and P-cystatin C that was elevated in one. No permanent AKI was diagnosed during the long-term follow-up of up to 32 months in any of these subjects.

### U-NGAL/crea

At baseline U-NGAL/crea was above the reference value (300 ng/g·crea, Fig. 1b) in one subject, 330 ng/ g·crea, with eGFR 59 mL/min/1,73m^2^. This patient had high U-NGAL/crea also at 4 h but normal ratio at 24 h. At 24 h none of the subjects had U-NGAL/crea above the reference value.

### U-KIM-1/crea

U-KIM-1/crea was above the reference value in two subjects both at baseline and at 24 h, their eGFR was moderately decreased 46 and 89 mL/min/1.73m^2^. At 24 h in five subjects U-KIM-1/crea was above the upper reference value (Fig. 1c). One of these five subjects had eGFR < 90 mL/min/1.73m^2^ at 24 h.

### U-IL-18 and U-L-FABP/crea

In total of 36 out of 90 samples U-IL-18 concentrations were above 25.6 ng/L (the lowest limit of quantification). Preoperatively, five of the subjects had U-IL-18 above the reference limit 65 pg/mL (22), one at 4 h and one subject at 24 h postoperatively (Fig. 1d).

Ten out of 90 measurements of U-L-FABP concentrations were higher than 6.25 μg/L (the lowest limit of quantification). At baseline two, at 4 h three subjects and at 24 h one subject had U-L-FABP/crea above the reference limit 8.4 μg/g·crea (Nickolas et al. 2012) .

### Pain

**Fig. 2:**
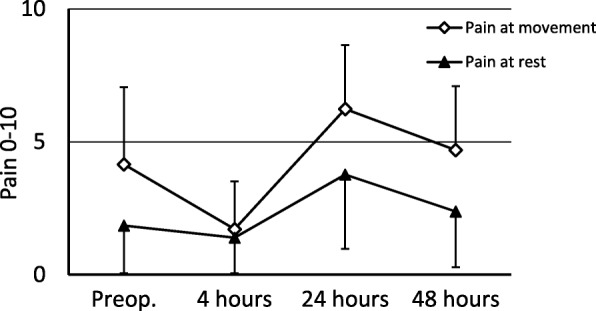
Pain ratings assessed with numerical rating scale (NRS, 0–10) preoperatively, at 4 h, 24 h and 48 h after surgery at rest and at leg movement. Data is presented as mean and standard deviation

A total of 25 subjects had used analgesics on a regular basis before surgery; acetaminophen (*n* = 6), NSAID (*n* = 2), acetaminophen and NSAID (*n* = 12), acetaminophen, NSAID and opioid (*n* = 3) or opioid and NSAID (n = 2). Pain ratings before and after surgery are presented in Fig. 2. All subjects needed oxycodone for rescue analgesia, the median dose for the first 48 postoperative hours was 91 (20–154) mg.

### Long-term outcome

Patients’ charts were examined between 28 and 32 months after surgery for nephrological diseases. There was no mortality during the follow-up. Plasma crea and eGFR was measured in 24 out of 30 subjects during the follow-up and median P-crea was 66 (38–95) μg/L and eGFR 84 (50–101) mL/kg/1.73 m^2^. Compared the values before surgery P-crea had increased in 14 and decreased in 6 subjects, the median change was 2 (− 14–18) μmol/L and eGFR had decreased in 16 subjects, median change − 2 (− 19–19) mL/kg/1.73 m^2^.

## Discussion

In the present study P-NGAL was the most often increased marker for renal deterioration. It was elevated in 28 of the 30 subjects, but only seven of these 28 subjects had P-crea, eGFR and P-cystatin C values within reference limits of the laboratory. Plasma NGAL is a marker of proximal tubular injury due to ischemia or toxic substances (Shavit et al. 2011), and it may differentiate kidney damage from rapidly corrected volume depletion (Nisula et al. 2015). In the present study the subjects were admitted for elective surgery, they were not volume depleted on admission and their hydration was controlled and treated. Thus, in these subjects elevated P-NGAL may have been a sign of minor proximal tubular injury.

In earlier studies P-NGAL has performed well in major surgery and critically ill patients (Schley et al. 2015; Kim et al. 2016). In the present study 28 subjects had elevated P-NGAL and 17 had low eGFR but normal P-crea. This supports the findings showing the insensitivity of P-crea to detect renal dysfunction when compared to eGFR (Swedko et al. 2003). A recent study shows that a combination of kidney function biomarkers, as in this study eGFR and P-NGAL, may detect early kidney deterioration in a timely manner in order to reduce complications like longer stay in hospital and higher mortality compared to patients without biomarkers’ rise (Haase et al. 2011).

According to literature increased U-NGAL with normal P-crea, would refer to less than 50% damage of renal mass or early detection of severe disease (Nickolas et al. 2012). If both U-NGAL and P-crea were increased, it would refer to damage to over 50% of renal mass (Nickolas et al. 2012). In the present study eight subjects had U-NGAL above 104 ng/mL, but their P-crea was normal, which would refer to early detection of AKI. However, the reference values for U-NGAL have not been established and the patient populations, elective surgical patients, and emergency unit triage patients (Nickolas et al. 2012), are different.

In the present study, five subjects developed transient, mild AKI defined by KDIGO criterion of urine output less than 0.5 mL/kg/h for the median time of 5.5 h they had a bladder catheter (Kellum et al. 2012). Urine output could be feasible indicator of early onset of AKI in orthopaedic patients and would lead to early recognition of the possible cause, e.g. nephrotoxic drugs or insufficient fluid input. In four of these five subjects P-NGAL was higher than 104 ng/mL. These high concentrations of P-NGAL possibly indicate subclinical tubular injury and support the assumption that P-NGAL could be a feasible biomarker in recognizing susceptible patients. However, there is no consensus of sensitive renal markers cut-off concentrations (Devarajan 2010). In previous studies comparing the performance, discriminative and predictive power of P- and U-NGAL in detecting AKI, almost opposite results have been reported (Schley et al. 2015; Fanning et al. 2016). However, some attempts to set reference values of P- and U-NGAL have been taken (Nickolas et al. 2012; Pennemans et al. 2013).

In the present study, the other novel renal biomarkers U-KIM-1, U-IL-18 and U-L-FABP did not provide additional information regarding renal deterioration. Urine KIM-1 did not correlate with P-crea, eGFR or P-cystatin C. In addition, both U-IL-18 and U-L-FABP  remained below the lower limit of quantification in most of the patients. Data about these biomarkers in patients undergoing orthopaedic surgery is sparse, but an earlier study in adult patients undergoing cardiac surgery has provided information that an increase in U-IL-18 and U-KIM-1 could be a prognostic factor for postoperative AKI and higher mortality (Parikh et al. 2013). In paediatric cardiac surgery increase in U-L-FABP was associated with risk for postoperative AKI undergoing cardiac surgery (Parikh et al. 2013).

Female gender, overweight, obesity and aging are known risk factors for knee osteoarthritis (Silverwood et al. 2015). This was seen also in the present study patients. Majority of them had also medical conditions, hypertension or diabetes and mild to moderate renal dysfunction that are known risk factors for postoperative AKI (Gharaibeh et al. 2017; Jämsä et al. 2017). Liberal use of NSAIDs may predispose these patients for further renal deterioration (Warth et al. 2016). In the present study P-crea did not reveal renal deterioration but in 17 of 30 subjects eGFR disclosed mildly to moderately decreased renal function. Although 57% of patients had mildly to moderately decreased renal function, all patients had LIA with ketorolac and 87% of patients were prescribed NSAIDs for postoperative analgesia. After LIA, ketorolac systemic exposure is similar than after intramuscular administration (Affas et al. 2014). Thus, avoiding NSAIDs in patients with pre-existing renal impairment should be considered in order to diminish the risk of postoperative AKI (Warth et al. 2016).

Different drug combinations are used in LIA. In their technique, Kerr and Kohan (2008) used ropivacaine, ketorolac and epinephrine. Since then, different local anaesthetics have been used and co-administered with corticosteroids, opioid analgesics and clonidine (Seangleulur et al. 2016; Ross et al. 2017). However, there is no consensus on the choice between various components to be used in LIA or which combination would have the best risk-benefit-ratio and for which patient groups.

Our study has some limitations. The number of participants was relatively small (*n* = 30) and without sample size calculations and control group. In addition, the patient cohort was heterogeneous considering their chronic diseases, and this may limit the interpretation of the data. However, the study was designed as a pilot study to test the feasibility of theses novel biomarkers available in the clinical patient population of the hospital. Lack of reference values of these novel renal biomarkers (Silverwood et al. 2015) and their prognostic value after orthopaedic surgery impair the utility of the data also. As NGAL may be elevated in inflammatory situations like sepsis and cardiopulmonary bypass surgery (Fanning et al. 2016), also the effect of surgical trauma on elevated P-NGAL in the present study can not be ruled out. However, as P-NGAL negatively correlated to eGFR decrease and no other signs or symptoms of infection or inflammation were noted in our patients, NGAL increase was considered of renal origin. As the planned future study would assess the renal effects of LIA and NSAIDs in TKA patients, it was necessary to test the feasibility of renal markers in the target population. However, P-NGAL was the only biomarker that showed correlation with decreased eGFR. Searching for ideal markers for AKI has been of interest for recent years, yet none have shown sufficient performance in clinical settings. This could be due to variation in clinical settings, biomarker assays, patient characteristics and baseline kidney function.

## Conclusions

In conclusion, if any of the traditional markers, P-crea, eGFR or P-cystatin C, was out of reference values, P-NGAL was elevated and was moderately correlated with decrease eGFR after TKA and LIA. Only seven of the 28 subjects with high P-NGAL had normal traditional markers’ values indicating that P-NGAL could be a feasible and sensitive enough for detection of AKI combined with eGFR and is thus, worth for further evaluation in orthopaedic patients.

## Abbreviations

AKI: Acute kidney injury
crea: Creatinine
eGFR: Calculated glomerular filtration rate
ELISA: Enzyme-linked-immuno-sorbent assay
h: Hour
HAVR: Hepatitis A virus cellular receptor 1
i.v.: Intravenous
IL-18: Interleukin-18
KDIGO: Kidney Disease Improving Global Outcomes
kim-1: Kidney injury molecule-1
L-FABP: Liver-type fatty acid-binding protein
LIA: Local infiltration analgesia
NGAL: Neutrophil gelatinase associated lipocalin
NRS: Numeric rating scale
NSAID: Nonsteroidal anti-inflammatory analgesic drugs
P: Plasma
r: Correlation coefficient
SPSS: Statistical Package for Social Science -software
TIM-1: T cell immunoglobulin and mucin domain-1
TKA: Total knee arthroplasty
U: Urine
